# N-Truncated Aβ Starting at Position Four—Biochemical Features, Preclinical Models, and Potential as Drug Target in Alzheimer’s Disease

**DOI:** 10.3389/fnagi.2021.710579

**Published:** 2021-08-20

**Authors:** Thomas A. Bayer

**Affiliations:** Division of Molecular Psychiatry, Department of Psychiatry and Psychotherapy, University Medical Center Göttingen (UMG), Georg-August-University, Göttingen, Germany

**Keywords:** N-truncated Aβ, Tg_4–42_, transgenic mouse model, immunotherapy, neuron loss, PET, *in vivo* imaging, memory decline

## Abstract

The discussion of whether amyloid plaque Aβ is a valid drug target to fight Alzheimer’s disease (AD) has been a matter of scientific dispute for decades. This question can only be settled by successful clinical trials and the approval of disease-modifying drugs. However, many clinical trials with antibodies against different regions of the amyloid Aβ peptide have been discontinued, as they did not meet the clinical endpoints required. Recently, passive immunization of AD patients with Donanemab, an antibody directed against the N-terminus of pyroglutamate Aβ, showed beneficial effects in a phase II trial, supporting the concept that N-truncated Aβ is a relevant target for AD therapy. There is long-standing evidence that N-truncated Aβ variants are the main variants found in amyloid plaques besides full-length Aβ_1–42_, t, therefore their role in triggering AD pathology and as targets for drug development are of interest. While the contribution of pyroglutamate Aβ_3–42_ to AD pathology has been well studied in the past, the potential role of Aβ_4–42_ has been largely neglected. The present review will therefore focus on Aβ_4–42_ as a possible drug target based on human and mouse pathology, *in vitro* and *in vivo* toxicity, and anti-Aβ_4-X_ therapeutic effects in preclinical models.

## Introduction

Even though the field of Alzheimer’s disease (AD) research has rapidly developed over the last decade, there is still a lack of disease-modifying therapies. Passive immunization with Donanemab a pyroglutamate Aβ (Aβ_pE3_) specific antibody showed disease-modification on cognition and for the ability to perform the activities of daily living (Mintun et al., 2021). No biomarkers are yet available based on N-truncated Aβ although autoantibodies were identified in plasma (Marcello et al., 2011). Trieb et al. (1996) investigated whether amyloid-β peptides may be relevant targets for the immune system using peripheral blood lymphocytes from healthy blood donors and patients with AD. While healthy donors elicited normal proliferative responsiveness after stimulation, a significant reduction was observed using lymphocytes from AD patients. Meanwhile, lower levels of naturally occurring anti-Aβ auto-antibodies have also been reported in the CSF (Du et al., 2001) and sera (Weksler et al., 2002) of AD patients, and elevated serum levels were also reported (Nath et al., 2003). It is also of note that in plasma of patients with mild cognitive impairment (MCI) and AD reduced pools of autoantibodies of the IgM class directed against pyroglutamate Aβ_3-X_ (Aβ_pE3-X_) have been reported (Marcello et al., 2009). In MCI patients, the level of the autoantibodies correlated with cognitive performance as evaluated by mini-mental state examination. N- and C-terminally truncated Aβ variants, their potential function and toxicity as well as their potential as drug targets were discussed recently (Bayer and Wirths, 2014; Dunys et al., 2018; Wirths and Zampar, 2019). The current mini-review, discusses the potential role of N-truncated Aβ in AD, focussing on N-truncated Aβ starting with position four Aβ_4–42_.

## Discovery of N-Truncated Aβ_4–42_ and Prevalence in The Human Brain

As revealed by a study by Portelius et al. (2010), the relative prevalence of full-length and N-truncated Aβ is of significant interest within the Alzheimer field. The authors used Aβ antibodies binding to Aβ_4–9_ and Aβ_8–22_ for immunoprecipitation. This was followed by mass spectrometry for identification of all Aβ variants in post-mortem tissue from patients with sporadic AD, familial AD with mutations in the presenilin-1 (PS-1; PSEN-1), or amyloid precursor protein (APP) genes. The authors demonstrated that the dominating Aβ isoforms are Aβ_1–42_, Aβ_pE3–42_, Aβ_4–42_, and Aβ_1–40_. The most prevalent variants in the hippocampus and the cortex were Aβ_1–42_ and Aβ_4–42_. The importance of Aβ_4–42_ did not receive appropriate attention in the past, although N-truncated Aβ_4-X_ has been discovered with the first sequencing endeavors of Aβ peptides isolated from plaque cores. This surprising finding puzzled Masters et al. (1985) as the most abundant variant of Aβ in the formic acid soluble fraction of plaque cores and subsequent peptide sequencing started with phenylalanine at position four (Aβ_4-X_) and not with the full-length Aβ_1-X_ they had hoped.

Glenner and Wong (Glenner and Wong, 1984) published ground-breaking work showing the full-length sequence of Ab peptides derived from the vasculature of AD patients. A number of reports have discussed the dominant presence of N-truncated Aβ variants within amyloid plaques in AD and Down syndrome patients (Harigaya et al., 2000; Tekirian, 2001; Miravalle et al., 2005; Piccini et al., 2005; Jawhar et al., 2011; Bayer and Wirths, 2014), while others suggest that full-length Aβ_1–42_ is pathologically relevant (Haass et al., 1992; Näslund et al., 1994; Selkoe, 2001; Walsh et al., 2002). Haass et al. (1993) discovered that Aβ is produced as a normal physiological process in APP transfected cell lines and cultured cells. The cells were analyzed by epitope mapping and radiosequencing of secreting Aβ variants. They observed mainly full-length Aβ_1–42_ (aspartate-1), but also two other Aβ peptides starting with the amino acid phenylalanine at position four and glutamate at position 11.

Although different methodologies for extracting and solubilizing aggregated Aβ have the potential for over- or under-estimating the relative prevalence of the various Aβ pools within amyloid plaques, there is general agreement that the C-terminus mostly ends with Aβ_X-42_ (alanine-42) and less abundantly with Aβ_X-40_ (arginin-40). For example, Ancolio et al. (1999) have demonstrated a large elevation of N-truncated Aβ_x–42_ in familial AD, postulating that all Aβ_x–42_ variants are the main factors driving AD pathology.

The idea that N-truncated Aβ may represent a potential drug target to fight AD has been largely neglected but was brought into attention recently (Jawhar et al., 2011; Bayer and Wirths, 2014; Cabrera et al., 2018). After this original discovery (Masters et al., 1985), other research groups have verified the presence of Aβ_4–42_ by other methodologies. Miller et al. (1993) employed matrix-assisted, laser-desorption-time-of-flight mass spectrometry of Aβ peptides isolated from plaque cores or the cerebrovasculature obtained from patients with AD. The authors demonstrated that the C-terminus of Aβ peptides within plaques ended with Aβ_42_, whereas cerebrovascular Aβ ended at Aβ_40_. They also proved that N-truncated Aβ_4-X_ represents a dominant fraction within plaque cores. Lewis et al. (2006) employed surface-enhanced laser desorption/ionization mass spectrometry for identifying the composition of Aβ peptides in AD and vascular dementia patients. In the brain of AD patients, Aβ started mostly with Phe-4, but other N-termini starting with Asp-1, Ala-2, pE-3, and Arg-5 were also detected in extractions from plaque cores. Using specific Aβ antibodies, Lemere et al. (1996) showed that patients with Down syndrome harbor Aβ starting at Asp-1 or pE-3 in a subset of plaques. Zampar et al. (2020) used capillary isoelectric focusing for showing Aβ antibody specificity of the N-terminus. Furthermore, the authors used immunohistochemistry with the verified N-terminal specific Aβ antibodies to stain post-mortem brain tissue from sporadic AD patients. They concluded that the staining signal for Aβ_1-X_ was much weaker in plaques as compared to cerebrovascular amyloid. In contrast, the signal for Aβ_4-X_ was much more evident in amyloid plaques.

Sergeant et al. (2003) verified that N-truncated Aβ represented the majority of all variants in both AD and pre-symptomatic AD with a substantial amount being Aβ_4–42_. The authors explored brain specimen from non-demented individuals with low amyloid load and tangle formation by Western blotting and mass spectrometry of the formic acid soluble fraction of amyloid plaques. Rosen et al. (2016) compared the composition of amyloid peptides isolated from AD neocortex and aged squirrel monkeys using immunochemical staining with an Aβ_4-X_ specific antibody and by mass spectrometry. The authors confirmed the high prevalence of N-truncated Aβ peptides including Aβ_4–42_ in the AD brain, while the prevalence in the non-human primate brain was low.

Although it has been shown that Aβ_4-X_ is generated and secreted *in vitro*, it was a matter of concern whether N-truncation and post-translational modifications of Aβ represent a post-mortem artifact due to long-term storage or tissue handling. Such an assumption can now be neglected with the therapeutic effect of an Aβ_pE3_-specific antibody in patients with AD after passive immunization (Mintun et al., 2021). Wildburger et al. (2017) employed high-resolution mass spectrometry to explore the question of whether N-truncation and other post-translational modifications of Aβ are found in AD brains due to post-mortem artifacts. The authors studied different Aβ pools depending on their solubility and concluded that the N-truncated variants did not correlate with post-mortem interval.

## Aβ_4-X_ Can Be Generated by Enzymatic Cleavage

**Figure 1:**
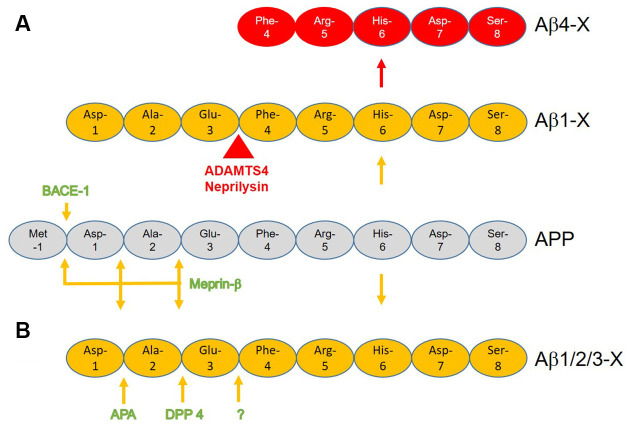
Schematic presentation of the potential generation of N-terminal Aβ_4-X_ with known enzymatic cleavages of the amyloid precursor protein (APP) and Aβ_1-X_. **(A)** β-site APP cleaving enzyme 1 (BACE-1) or meprin-β cuts APP between amino acid position -1 and +1 liberating the N-terminus of full-length Aβ_1-X_. While ADAMTS4 further cuts between position +3 and +4 liberating Aβ_4–40/42_, the normal function of neprilysin is to detoxify Aβ as it has several N-terminal activities within Aβ with N-truncation of Aβ_4-X_ being only one alternative. **(B)** Hypothetical pathway(s) by which N-truncated Aβ_4-x_ is generated by sequential cleavage by meprin-β, aminopeptidase A (APA), and/or dipeptidyl peptidase 4 (DPP 4).

The generation of N-terminal Aβ_4-X_ by known enzymatic cleavages has been reviewed before in detail (Bayer and Wirths, 2014). It can be generated by a two-step process starting with β-site APP cleaving enzyme 1 (BACE-1) cutting APP between Met at postion-1 and Asp at position +1 liberating the N-terminus of full-length Aβ_1-X_ (Vassar et al., 1999). ADAMTS4 (a disintegrin and metalloproteinase with thrombospondin motifs 4) and neprilysin (NEP) further cuts between Glu at position +3 and Phe at position +4 liberating the N-terminus of Aβ_4-X_ (Bayer and Wirths, 2014; Walter et al., 2019; Figure 1A). NEP cleaves Aβ at multiple sites thereby detoxifying amyloid-β peptides (Bayer and Wirths, 2014). Walter et al. (2019) identified a recognition site for the secreted form of metalloprotease ADAMTS4 within the full-length Aβ sequence. The induction of ADAMTS4 expression in cell culture led to increased secretion of Aβ_4–40_ the levels of Aβ_1-x_ were not altered. Furthermore, the authors identified adult oligodendrocytes as the only source of ADAM4TS triggered Aβ_4-X_ generation in the murine brain. The main function of NEP on Aβ is degradation and catabolism of the peptide (Iwata et al., 2001; Leissring et al., 2003). The loss of NEP activity leads to enhanced levels of brain and plasma levels of full-length Aβ, the elevated half-life of soluble Aβ, and increased amyloid plaque pathology in the J9 mouse model of AD (Farris et al., 2007). Hama et al. (2004) showed that different intracellular compartments are involved in the degradation of amyloid peptides by NEP. Thus NEP is mainly responsible for the intra- and extracellular neuronal clearance of Aβ peptides and more importantly also at the presynaptic site (Iwata et al., 2004). Elevated NEP impaired hippocampal synaptic plasticity and cognitive function in the APP23 mouse model for AD (Huang et al., 2006). Besides general clearance of full-length Aβ peptides, NEP is also involved in the generation of N-truncated Aβ peptides Aβ_4-X_ (Bayer and Wirths, 2014), and further clearing of Aβ_4–42_ *in vivo* and *in vitro* (Hornung et al., 2019). Aβ_4–9_, a main degradation product of NEP, is a major Cu^2+^ binding and has been suggested as a possible Cu^2+^ carrier in the brain (Bossak-Ahmad et al., 2019) and NEP modulation (Mital et al., 2018). Modulating Cu metabolism is discussed as a relevant therapeutic target (Lei et al., 2020). In APP23 mice, Cu supplementation lowered amyloid plaque load and stabilized Cu-dependent superoxide dismutase-1 activity (Bayer et al., 2003). In mild to moderate AD patients cognitive decline correlated with low plasma concentrations of Cu (Pajonk et al., 2005). However, treatment for 12 months with supplemental Cu had no effect on cognition in patients with mild AD in a phase 2 clinical trial (Kessler et al., 2008). Alternatively, N-truncated Aβ_4-X_ may be generated directly by cutting APP between Glu at position 3 and Phe at position 4 by unknown enzymatic activity. Of note, N-truncated Aβ_4-X_ is secreted together with Aβ_1–42_ in APP overexpressing cells *in vitro*, which may indicate unknown enzymatic activity in neurons (Haass et al., 1993). For example, N-truncated Aβ_5-X_ is mainly produced from the caspase-cleaved form of APP and not from full-length Aβ (Murayama et al., 2007). Another potential alternative may be the generation the Aβ4-x peptide, a sequential cleavage of full-length Aβ by aminopeptidase A, meprin-β or dipeptidyl-peptidases (Sevalle et al., 2009; Antonyan et al., 2018; Schlenzig et al., 2018; Valverde et al., 2021). At least theoretically, Aβ_4-x_ could be derived from Aβ_2-x_ or Aβ_3-x_ peptide (Figure 1B).

## Acute Effect of N-Truncated Aβ_4–42_

Exposure of soluble oligomers of Aβ_4–42_ preparations induced neuron degeneration after 7 days of cultured rat primary cortical neurons (Antonios et al., 2013, 2015). Injecting Aβ_4–42_ into the lateral ventricles of wildtype mice induced working memory deficits after 4 days (Antonios et al., 2013, 2015). In both assays, the neurodegenerative effect of Aβ_4–42_ was similar to the exposure of Aβ_1–42_ and Aβ_pE3–42_. In 1995, Pike et al. (1995) claimed that Aβ peptides with N-terminal truncations including Aβ_4-X_ exhibited enhanced peptide aggregation relative to full-length Aβ species. Furthermore, they concluded from obtained CD (circular dichroism) spectra that β-sheets were the predominantly formed conformations by all Aβ variants. They exhibited fibrillary morphology viewed by transmission electron microscopy, and induced degeneration of cultured rat hippocampus neurons. Bouter et al. (2013) demonstrated that soluble oligomeric aggregates derived from Aβ_4–42_ and Aβ_pE3–42_ have specific structural features distinct from full-length Aβ_1–42_, although fibril formation as reported previously (Pike et al., 1995) was comparable. Spectral alterations using ultraviolet circular dichroism spectroscopy showed that Aβ_4–42_ forms a folded conformation upon heating (Bouter et al., 2013). Using the liquid state nuclear magnetic resonance technique Aβ_4–42_ and Aβ_pE3–42_ exhibited soluble and stable aggregates, which was less pronounced for Aβ_1–42_. However, the size of Aβ_4–42_ and Aβ_pE3–42_ aggregates were different from those formed by Aβ_1–42_ (Bouter et al., 2013). Even though Aβ_1–42_, Aβ_pE3–42_ and Aβ_4–42_ are unstructured in the monomeric state. After heating all Aβ peptides formed of folded structures. Interestingly, monomeric Aβ_4–42_ and Aβ_pE3–42_ rapidly converted into soluble oligomeric forms in contrast to full-length Aβ_1–42,_ which stay in equilibrium for a longer time between monomers and oligomers (Bouter et al., 2013).

Parodi-Rullan et al. (2020) studied the effect of full-length and different N-terminal truncated Aβ variants on blood-brain barrier permeability, cerebral microvascular endothelial cell viability, and angiogenesis. The authors demonstrated that Aβ_4–42_ followed by Aβ_4–40_ was the most potent inhibitor of angiogenesis, and they were also much stronger than Aβ_1–42_ and Aβ_1–40_.

## Chronic Effect of N-Truncated Aβ_4–42_

The development of the APP/PSEN-1 double-transgenic mouse model, APP/PS1KI, for AD revealed, besides massive neuron loss in the hippocampus, many N-truncated Aβ_X-42_ variants including Aβ_4–42_ elucidated by two-dimensional Western blotting, which were subsequently verified by mass spectrometry (Casas et al., 2004). The 5XFAD mouse model is more widely used in the scientific community expressing mutant APP and PSEN-1 transgenes (Oakley et al., 2006). Mass spectrometric analysis with N-terminal specific Aβ antibodies of 5XFAD mouse brain elucidated that the vast majority of Aβ peptides were full-length Aβ_1–42_ (Wittnam et al., 2012).

Using a highly specific antiserum against the N-terminus of Aβ_4-X_ abundant plaque staining was observed in APP/PS1KI and 5XFAD transgenic mouse brain (Wirths et al., 2017). In the human AD brain, this antiserum demonstrated staining of plaque cores of senile plaques, but none in diffuse amyloid deposits. The peptide content of plaques from AD brain and an AD mouse model (presenilin-2/APP transgenic mice, PS2APP) was analyzed using laser dissection microscopy combined with mass spectrometry (Rufenacht et al., 2005). The authors described various N-terminal truncated Aβ peptides in PS2APP and AD amyloid plaques however with significantly elevated levels in AD brain.

Kawarabayashi et al. (2001) studied another AD mouse model with mutant APP (Tg2576) and elucidated that only a minor fraction of Aβ was N-terminally truncated in contrast to AD brain (Kawarabayashi et al., 2001). This was verified, by another study by Kalback et al. (2002). The authors employed size-exclusion and reverse-phase chromatography, amino acid sequencing, and mass spectrometry of amyloid plaques of Tg2576 mice. The authors found that the amyloid plaques differed in their physical and chemical properties from those isolated from the AD brain. In Tg2576 mice, most peptides were full-length Aβ_1–42_, whereas, in AD brains, most peptides were N-truncated. The brain tissue of the transgenic mouse models mentioned above were freshly prepared without any post-mortem delay, therefore changes in pH, long-storage artifacts, state of agony, and medication, etc. can be ruled out as an explanation of the appearance of N-truncated Aβ variants in transgenic mouse brain.

**Figure 2:**
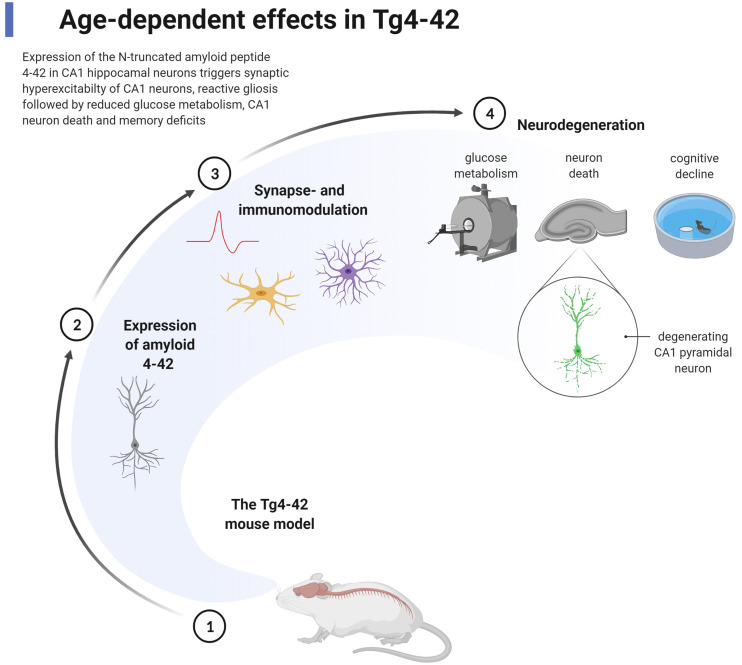
Chronic effect of the expression of Aβ_4–42_ in the Tg4–42 mouse model for Alzheimer’s disease (AD). The transgenic mouse model Tg4–42 (1) expresses exclusively normal wildtype human Aβ_4–42_ predominantly in pyramidal neurons in the CA1 area of the hippocampus (2). The expression of Aβ_4–42_ induces early (starting at 2–3 months of age) pathological effects highlighted by synaptic hyperexcitability of CA1 pyramidal neurons, reactive micro- and astroglia (3). As a consequence of chronic exposure of Aβ_4–42_ (4), massive reduction in glucose metabolism is detected by ^18^F-PET/magnetic resonance imaging (MRI), loss of degenerating CA1 pyramidal neurons, and loss of spatial reference memory analyzed by the Morris water maze test (starting at 4–6 months of age). The figure shows methodologies in addition to pathological events using appropriate symbols. Created with BioRender.com.

The Tg4–42 mouse model for AD expresses only Aβ_4–42_, which represents a unique model system for studying the effect of chronic exposure of Aβ_4–42_ in the mouse brain (Bouter et al., 2013; Figure 2). The long-lasting exposure of Aβ_4–42_ induced an age-dependent neuron loss in the hippocampus, which correlated with hippocampus-dependent spatial reference memory deficits. Tg4–42 mice demonstrated synaptic hyper-excitability, changes in short-term synaptic plasticity but no effects on short- and long-term potentiation in the hippocampus (Dietrich et al., 2018). Busche et al. (2012) have demonstrated that full-length Aβ induced neuronal hyperactivity in brain slice cultures of wild-type mice. Synaptic hyperactivity of hippocampal pyramidal neurons is therefore an early event in AD pathology. ^18^F-Fluorodeoxyglucose (^18^F-FDG)-PET in combination with magnetic resonance imaging (MRI) in Tg4–42 mice was used for analyzing cerebral brain glucose metabolism *in vivo* (Hinteregger et al., 2021). Tg4–42 mice demonstrated lower glucose uptake correlating with neuron loss and memory deficits in an age-dependent manner (Bouter et al., 2018). The reduction of glucose metabolism was detected already in young Tg4–42 prior to neuron death and neurological deficits. In clinical settings, the quantification of brain glucose uptake using ^18^F-FDG-PET is an established diagnostic tool widely used for differential diagnosis of patients with dementia, including AD (Chetelat et al., 2020). The expert panel suggested a diagnostic algorithm with appropriate time-points using amyloid-PET and ^18^F-FDG-PET for better clinical management of patients with AD. ^18^F-FDG-PET signal is a powerful tool to monitor cerebral glucose consumption *in vivo*, a measure for synaptic activity (Sokoloff, 2008). It is also a valuable tool in preclinical research (Bouter and Bouter, 2019). A comparison between 5XFAD and Tg4–42 showed, that only young Tg4–42 developed robust neurological deficits, whereas in aged mice both models elicited similar memory deficits and fear conditioning tasks (Bouter et al., 2014). Reduced acoustic startle response, prepulse inhibition, and motor coordination were reported in Tg4–42 (Sichler et al., 2019; Wagner et al., 2019). Differentially expressed mRNAs in the brain of Tg4–42 and 5XFAD were identified by deep sequencing. A significant number of mRNAs were associated with memory deficits and neuron loss, which points to common disease pathways in both AD models (Bouter et al., 2014). Moreover, small RNA sequencing of the microRNAome in the hippocampus of Tg4–42 mice revealed microRNAs involved in learning, memory function, and synaptic signaling (Bouter et al., 2020). Metabolic changes of the glutamate/4-aminobutyrate-glutamine axis correlated with neurological deficits, neurodegeneration, and elevated CSF levels of neurofilament light chain in aged Tg4–42 mice (Hinteregger et al., 2021). Tg4–42 mice harbor more than 20 copies of the transgene in exon 2 of the retinoic acid receptor β (RARB) leading to decreased expression of RARB, which could have an (at least partial) effect on the phenotype (Hinteregger et al., 2020). Co-expression of Aβ_4–42_ with Aβ_pE3–42_ within the same neuron accelerated neurodegeneration in the hippocampus, enhanced loss of anxiety and motor deficits as well as sensori-motor deficits (Lopez-Noguerola et al., 2018).

## N-Truncated Aβ_4-X_ as The Target for Immunotherapy

The discovery by Schenk et al. (1999) represents an important development in the AD field because it was the first time that disease-modulation was shown to be possible. The authors showed that pre-aggregated synthetic Aβ_1–42_ drastically reduced amyloid plaques together with associated astrogliosis in the PDAPP transgenic mouse model for AD. This observation was supported by a subsequent report by Morgan et al. (2000) using another transgenic mouse model for AD (a cross of the Tg2576 and the PS1M146L transgenic line (Holcomb et al., 1998), demonstrating that memory decline assessed by the radial-arm water-maze test was stabilized by active immunization with full-length Aβ.

Janus et al. (2000) observed similar treatment effects of vaccination with Aβ_1–42_ in the TgCRND8 model. This was followed by exploring the effect of active immunization with AN1792 (pre-aggregated full-length Aβ_1–42_) in patients with AD. In phase I and IIa clinical trials a subset of the patients developed aseptic meningoencephalopathy and was therefore discontinued despite some hints that it might stabilize cognitive decline in a small subset of patients (Hock et al., 2002; reviewed in Morgan, 2011). Boche et al. (2008) demonstrated that amyloid plaques are solubilized by anti-Aβ antibodies after active immunization with pre-aggregated full-length Aβ_1–42_. Consequently, the solubilized amyloid-β is drained *via* the perivascular pathway and was found to be increased in the brain.

Passive immunization with antibodies against Aβ had beneficial treatment effects in AD mouse models and is, therefore, another promising approach to modulating amyloid pathology *in vivo*. Demattos et al. (2001) used the monoclonal antibody m266 directed against the central domain of Aβ for passive immunization of PDAPP mice leading to reduced amyloid load. Stabilizing memory deficits using an object recognition task and a holeboard learning and memory task without a treatment effect on amyloid load in PDAPP mice was also reported (Dodart et al., 2002). However, Solanezumab the humanized version of m266 did not reach clinical endpoints on cognitive or functional abilities in phase III clinical trials with AD patients (Doody et al., 2014). In clinical phase III trials, bapineuzumab, the humanized monoclonal antibody derived from murine 3D6 was directed against Aβ_1–5_ (Johnson-Wood et al., 1997) and did not improve clinical outcomes in patients with AD (Salloway et al., 2014). A comprehensive review of other antibody candidates for the treatment of AD has entered clinical trials and novel drug targets have recently been discussed in detail by Cummings et al. (2020).

Mclaurin et al. (2002) reported that active immunization with the full-length Aβ_1–42_ peptide of TgCRND8 mice reduced amyloid plaques and rescued memory deficits. Interestingly, the therapeutically active antibodies in this experimental setup recognized residues 4–10 of Aβ_1–42_, an indication that N-truncated Aβ_4-X_ may be a relevant drug target. The therapeutic active anti-Aβ_4–10_ antibodies also influenced aggregation propensity and toxicity *in vitro*. We have generated novel murine monoclonal Aβ antibodies against N-truncated Aβ_4-X_ (and Aβ_pE3-X_) using preparations of freshly prepared Aβ_4–40_ and used the antibody NT4X for further studies (Antonios et al., 2013, 2015). NT4X rescued the acute toxic effects of Aβ_4–42_ using rat primary neurons and *in vivo* by cerebroventricular injection into wildtype mice (Antonios et al., 2013). NT4X also rescued the chronic effects of Aβ_4–42_ on pyramidal neuron loss in the hippocampus and spatial reference memory deficits after passive immunization (Antonios et al., 2015). Therefore, besides Aβ_pE3–42,_ Aβ_4–42_ might be another relevant drug target in AD.

The question of whether N-truncations of Aβ within plaques represent a post-mortem artifact or might even precede the symptomatology of AD was addressed by Russo et al. (1997), demonstrating that both Aβ_1-X_ and Aβ_pE-X_ can form stable water-soluble aggregates not related to aggregates amyloid within plaques. Moreover, Rijal Upadhaya et al. (2014) studied post-mortem brain tissue from AD cases with symptomatic and preclinical AD by Western blot and demonstrated that Aβ_pE3-X_ is an informative biomarker for biochemical amyloid-β staging. Aβ_pE-X_ and a phosphorylated Aβ variant were not only detectable in plaques but also in soluble aggregates. The authors concluded that the different Aβ variants occur in a hierarchical sequence that allows the distinction of three stages, and may therefore be relevant for therapeutic intervention (Rijal Upadhaya et al., 2014). Finally, Mintun et al. (2021) conducted a phase 2 clinical trial of Donanemab in patients with early symptomatic AD, an antibody that specifically detects Aβ_pE3-X_ in plaques. The patients were selected based on the amount of tau and amyloid deposition on positron-emission tomography. The outcome of the study showed that a group of patients had a significantly better cognitive score than the placebo group as well as lower amyloid and tau load.

## Author Contributions

TB wrote the article and designed the figures.

## Conflict of Interest

The University Medicine of Göttingen holds patents, has submitted patent applications on the research tools as well as therapeutic antibodies against N-truncated Aβ. The author declares that the research was conducted in the absence of any commercial or financial relationships that could be construed as a potential conflict of interest.

## Publisher’s Note

All claims expressed in this article are solely those of the authors and do not necessarily represent those of their affiliated organizations, or those of the publisher, the editors and the reviewers. Any product that may be evaluated in this article, or claim that may be made by its manufacturer, is not guaranteed or endorsed by the publisher.

## References

[B1] Ancolio K. Dumanchin C. Barelli H. Warter J. M. Brice A. Campion D.. (1999). Unusual phenotypic alteration of β amyloid precursor protein (β APP) maturation by a new Val715Met β APP-770 mutation responsible for probable early-onset alzheimer’s disease. Proc. Natl. Acad. Sci. U S A 96, 4119–4124. 10.1073/pnas.96.7.4119 10097173 PMC22430

[B2] Antonios G. Borgers H. Richard B. C. Brauss A. Meissner J. Weggen S.. (2015). Alzheimer therapy with an antibody against N-terminal Aβ 4-X and pyroglutamate Aβ 3-X. Sci. Rep. 5:17338. 10.1038/srep17338 26626428 PMC4667289

[B3] Antonios G. Saiepour N. Bouter Y. Richard B. C. Paetau A. Verkkoniemi-Ahola A.. (2013). N-truncated Aβ starting with position four: early intraneuronal accumulation and rescue of toxicity using NT4X-167, a novel monoclonal antibody. Acta Neuropathol. Commun. 1:56. 10.1186/2051-5960-1-56 24252153 PMC3893517

[B4] Antonyan A. Schlenzig D. Schilling S. Naumann M. Sharoyan S. Mardanyan S.. (2018). Concerted action of dipeptidyl peptidase IV and glutaminyl cyclase results in formation of pyroglutamate-modified amyloid peptides *in vitro*. Neurochem. Int. 113, 112–119. 10.1016/j.neuint.2017.12.001 29224965

[B5] Bayer T. A. Wirths O. (2014). Focusing the amyloid cascade hypothesis on N-truncated Aβ peptides as drug targets against Alzheimer’s disease. Acta Neuropathol. 127, 787–801. 10.1007/s00401-014-1287-x 24803226 PMC4024135

[B6] Bayer T. A. Schafer S. Simons A. Kemmling A. Kamer T. Tepest R.. (2003). Dietary Cu stabilizes brain superoxide dismutase 1 activity and reduces amyloid Aβ production in APP23 transgenic mice. Proc. Natl. Acad. Sci. U S A 100, 14187–14192. 10.1073/pnas.2332818100 14617773 PMC283567

[B7] Boche D. Zotova E. Weller R. O. Love S. Neal J. W. Pickering R. M.. (2008). Consequence of Aβ immunization on the vasculature of human Alzheimer’s disease brain. Brain 131, 3299–3310. 10.1093/brain/awn261 18953056

[B8] Bossak-Ahmad K. Mital M. Plonka D. Drew S. C. Bal W. (2019). Oligopeptides generated by neprilysin degradation of β-amyloid have the highest Cu(II) affinity in the whole Aβ family. Inorg. Chem. 58, 932–943. 10.1021/acs.inorgchem.8b03051 30582328

[B9] Bouter C. Bouter Y. (2019). (18)F-FDG-PET in mouse models of Alzheimer’s disease. Front. Med. (Lausanne) 6:71. 10.3389/fmed.2019.00071 31058151 PMC6482246

[B10] Bouter C. Henniges P. Franke T. N. Irwin C. Sahlmann C. O. Sichler M. E.. (2018). (18)F-FDG-PET detects drastic changes in brain metabolism in the Tg4–42 model of Alzheimer’s disease. Front. Aging Neurosci. 10:425. 10.3389/fnagi.2018.00425 30670962 PMC6333025

[B11] Bouter Y. Dietrich K. Wittnam J. L. Rezaei-Ghaleh N. Pillot T. Papot-Couturier S.. (2013). N-truncated amyloid β (Aβ) 4–42 forms stable aggregates and induces acute and long-lasting behavioral deficits. Acta Neuropathol. 126, 189–205. 10.1007/s00401-013-1129-2 23685882 PMC3722453

[B12] Bouter Y. Kacprowski T. Rossler F. Jensen L. R. Kuss A. W. Bayer T. A.. (2020). miRNA alterations elicit pathways involved in memory decline and synaptic function in the hippocampus of aged Tg4–42 mice. Front. Neurosci. 14:580524. 10.3389/fnins.2020.580524 33013313 PMC7511553

[B13] Bouter Y. Kacprowski T. Weissmann R. Dietrich K. Borgers H. Brauss A.. (2014). Deciphering the molecular profile of plaques, memory decline and neuron loss in two mouse models for Alzheimer’s disease by deep sequencing. Front. Aging Neurosci. 6:75. 10.3389/fnagi.2014.00075 24795628 PMC3997018

[B14] Busche M. A. Chen X. Henning H. A. Reichwald J. Staufenbiel M. Sakmann B.. (2012). Critical role of soluble amyloid-β for early hippocampal hyperactivity in a mouse model of Alzheimer’s disease. Proc. Natl. Acad. Sci. U S A 109, 8740–8745. 10.1073/pnas.1206171109 22592800 PMC3365221

[B15] Cabrera E. Mathews P. Mezhericher E. Beach T. G. Deng J. Neubert T. A.. (2018). Aβ truncated species: Implications for brain clearance mechanisms and amyloid plaque deposition. Biochim. Biophys. Acta Mol. Basis Dis. 1864, 208–225. 10.1016/j.bbadis.2017.07.005 28711595 PMC5875988

[B16] Casas C. Sergeant N. Itier J. M. Blanchard V. Wirths O. Van Der Kolk N.. (2004). Massive CA1/2 neuronal loss with intraneuronal and N-terminal truncated Aβ 42 accumulation in a novel Alzheimer transgenic model. Am. J. Pathol. 165, 1289–1300. 10.1016/s0002-9440(10)63388-3 15466394 PMC1618627

[B17] Chetelat G. Arbizu J. Barthel H. Garibotto V. Law I. Morbelli S.. (2020). Amyloid-PET and (18)F-FDG-PET in the diagnostic investigation of Alzheimer’s disease and other dementias. Lancet Neurol. 19, 951–962. 10.1016/S1474-4422(20)30314-8 33098804

[B18] Cummings J. Lee G. Ritter A. Sabbagh M. Zhong K. (2020). Alzheimer’s disease drug development pipeline: 2020. Alzheimers Dement. (N Y) 6:e12050. 10.1002/trc2.12050 32695874 PMC7364858

[B19] Demattos R. B. Bales K. R. Cummins D. J. Dodart J. C. Paul S. M. Holtzman D. M.. (2001). Peripheral anti-Aβ antibody alters CNS and plasma Aβ clearance and decreases brain Aβ burden in a mouse model of Alzheimer’s disease. Proc. Natl. Acad. Sci. U S A 98, 8850–8855. 10.1073/pnas.151261398 11438712 PMC37524

[B20] Dietrich K. Bouter Y. Muller M. Bayer T. A. (2018). Synaptic alterations in mouse models for Alzheimer disease-A special focus on N-truncated Aβ 4–42. Molecules 23:718. 10.3390/molecules23040718 29561816 PMC6017701

[B21] Dodart J. C. Bales K. R. Gannon K. S. Greene S. J. Demattos R. B. Mathis C.. (2002). Immunization reverses memory deficits without reducing brain Aβ burden in Alzheimer’s disease model. Nat. Neurosci. 5, 452–457. 10.1038/nn842 11941374

[B22] Doody R. S. Thomas R. G. Farlow M. Iwatsubo T. Vellas B. Joffe S.. (2014). Phase 3 trials of solanezumab for mild-to-moderate Alzheimer’s disease. N. Engl. J. Med. 370, 311–321. 10.1056/NEJMoa1312889 24450890

[B23] Du Y. Dodel R. Hampel H. Buerger K. Lin S. Eastwood B.. (2001). Reduced levels of amyloid β-peptide antibody in Alzheimer disease. Neurology 57, 801–805. 10.1212/wnl.57.5.801 11552007

[B24] Dunys J. Valverde A. Checler F. (2018). Are N- and C-terminally truncated Aβ species key pathological triggers in Alzheimer’s disease? J. Biol. Chem. 293, 15419–15428. 10.1074/jbc.R118.003999 30143530 PMC6177599

[B25] Farris W. Schutz S. G. Cirrito J. R. Shankar G. M. Sun X. George A.. (2007). Loss of neprilysin function promotes amyloid plaque formation and causes cerebral amyloid angiopathy. Am. J. Pathol. 171, 241–251. 10.2353/ajpath.2007.070105 17591969 PMC1941603

[B26] Glenner G. G. Wong C. W. (1984). Alzheimer’s disease: Initial report of the purification and characterization of a novel cerebrovascular amyloid protein. Biochem. Biophys. Res. Commun. 120, 885–890. 10.1016/s0006-291x(84)80190-4 6375662

[B27] Haass C. Hung A. Y. Schlossmacher M. G. Oltersdorf T. Teplow D. B. Selkoe D. J.. (1993). Normal cellular processing of the β-amyloid precursor protein results in the secretion of the amyloid β peptide and related molecules. Ann. N Y Acad. Sci. 695, 109–116. 10.1111/j.1749-6632.1993.tb23037.x 8239267

[B28] Haass C. Schlossmacher M. G. Hung A. Y. Vigo Pelfrey C. Mellon A. Ostaszewski B. L.. (1992). Amyloid β-peptide is produced by cultured cells during normal metabolism. Nature 359, 322–325. 10.1038/359322a0 1383826

[B29] Hama E. Shirotani K. Iwata N. Saido T. C. (2004). Effects of neprilysin chimeric proteins targeted to subcellular compartments on amyloid-β peptide clearance in primary neurons. J. Biol. Chem. 279, 30259–30264. 10.1074/jbc.M401891200 15100223

[B30] Harigaya Y. Saido T. C. Eckman C. B. Prada C. M. Shoji M. Younkin S. G.. (2000). Amyloid β protein starting pyroglutamate at position 3 is a major component of the amyloid deposits in the Alzheimer’s disease brain. Biochem. Biophys. Res. Commun. 276, 422–427. 10.1006/bbrc.2000.3490 11027491

[B31] Hinteregger B. Loeffler T. Flunkert S. Neddens J. Bayer T. A. Madl T.. (2021). Metabolic, phenotypic and neuropathological characterization of the Tg4–42 mouse model for Alzheimer’s disease. J. Alzheimers Dis. 80, 1151–1168. 10.3233/JAD-201204 33646155 PMC8150512

[B32] Hinteregger B. Loeffler T. Flunkert S. Neddens J. Birner-Gruenberger R. Bayer T. A.. (2020). Transgene integration causes RARB downregulation in homozygous Tg4–42 mice. Sci. Rep. 10:6377. 10.1038/s41598-020-63512-8 32286473 PMC7156671

[B33] Hock C. Konietzko U. Papassotiropoulos A. Wollmer A. Streffer J. Von Rotz R. C.. (2002). Generation of antibodies specific for β-amyloid by vaccination of patients with Alzheimer disease. Nat. Med. 8, 1270–1275. 10.1038/nm783 12379846

[B34] Holcomb L. Gordon M. N. Mcgowan E. Yu X. Benkovic S. Jantzen P.. (1998). Accelerated Alzheimer-type phenotype in transgenic mice carrying both mutant amyloid precursor protein and presenilin 1 transgenes. Nat. Med. 4, 97–100. 10.1038/nm0198-097 9427614

[B35] Hornung K. Zampar S. Engel N. Klafki H. Liepold T. Bayer T. A.. (2019). N-terminal truncated Aβ 4–42 Is a substrate for neprilysin degradation *in vitro* and *in vivo*. J. Alzheimers Dis. 67, 849–858. 10.3233/JAD-181134 30664509

[B36] Huang S.-M. Mouri A. Kokubo H. Nakajima R. Suemoto T. Higuchi M.. (2006). Neprilysin-sensitive synapse-associated Aβ oligomers impair neuronal plasticity and cognitive function. J. Biol. Chem. 281, 17941–17951. 10.1074/jbc.M601372200 16636059

[B37] Iwata N. Mizukami H. Shirotani K. Takaki Y. Muramatsu S.-I. Lu B.. (2004). Presynaptic localization of neprilysin contributes to efficient clearance of amyloid-β peptide in mouse brain. J. Neurosci. 24, 991–998. 10.1523/JNEUROSCI.4792-03.2004 14749444 PMC6729819

[B38] Iwata N. Tsubuki S. Takaki Y. Shirotani K. Lu B. Gerard N. P.. (2001). Metabolic regulation of brain Aβ by neprilysin. Science 292, 1550–1552. 10.1126/science.1059946 11375493

[B39] Janus C. Pearson J. Mclaurin J. Mathews P. M. Jiang Y. Schmidt S. D.. (2000). A β peptide immunization reduces behavioural impairment and plaques in a model of Alzheimer’s disease. Nature 408, 979–982. 10.1038/35050110 11140685

[B40] Jawhar S. Wirths O. Bayer T. A. (2011). Pyroglutamate Aβ - a hatchet man in Alzheimer disease. J. Biol. Chem. 286, 38825–38832. 10.1074/jbc.R111.288308 21965666 PMC3234707

[B41] Johnson-Wood K. Lee M. Motter R. Hu K. Gordon G. Barbour R.. (1997). Amyloid precursor protein processing and A β42 deposition in a transgenic mouse model of Alzheimer disease. Proc. Natl. Acad. Sci. U S A 94, 1550–1555. 10.1073/pnas.94.4.1550 9037091 PMC19829

[B42] Kalback W. Watson M. D. Kokjohn T. A. Kuo Y. M. Weiss N. Luehrs D. C.. (2002). APP transgenic mice Tg2576 accumulate Aβ peptides that are distinct from the chemically modified and insoluble peptides deposited in Alzheimer’s disease senile plaques. Biochemistry 41, 922–928. 10.1021/bi015685+ 11790115

[B43] Kawarabayashi T. Younkin L. Saido T. Shoji M. Ashe K. Younkin S.. (2001). Age-dependent changes in brain, CSF and plasma amyloid (β) protein in the Tg2576 transgenic mouse model of Alzheimer’s disease. J. Neurosci. 21, 372–381. 10.1523/JNEUROSCI.21-02-00372.2001 11160418 PMC6763819

[B44] Kessler H. Bayer T. A. Bach D. Schneider-Axmann T. Supprian T. Herrmann W.. (2008). Intake of copper has no effect on cognition in patients with mild Alzheimer’s disease: a pilot phase 2 clinical trial. J. Neural Transm. 115, 1181–1187. 10.1007/s00702-008-0080-1 18587525 PMC2516533

[B45] Lei P. Ayton S. Bush A. I. (2020). The essential elements of Alzheimer’s disease. J. Biol. Chem. 296:100105. 10.1074/jbc.REV120.008207 33219130 PMC7948403

[B46] Leissring M. A. Farris W. Chang A. Y. Walsh D. M. Wu X. Sun X.. (2003). Enhanced proteolysis of β-amyloid in APP transgenic mice prevents plaque formation, secondary pathology and premature death. Neuron 40, 1087–1093. 10.1016/s0896-6273(03)00787-6 14687544

[B47] Lemere C. A. Blusztajn J. K. Yamaguchi H. Wisniewski T. Saido T. C. Selkoe D. J.. (1996). Sequence of deposition of heterogeneous amyloid β-peptides and APO E in Down syndrome: implications for initial events in amyloid plaque formation. Neurobiol. Dis. 3, 16–32. 10.1006/nbdi.1996.0003 9173910

[B48] Lewis H. Beher D. Cookson N. Oakley A. Piggott M. Morris C. M.. (2006). Quantification of Alzheimer pathology in ageing and dementia: age-related accumulation of amyloid-β (42) peptide in vascular dementia. Neuropathol. Appl. Neurobiol. 32, 103–118. 10.1111/j.1365-2990.2006.00696.x 16599940

[B49] Lopez-Noguerola J. S. Giessen N. M. E. Ueberuck M. Meissner J. N. Pelgrim C. E. Adams J.. (2018). Synergistic effect on neurodegeneration by N-truncated Aβ 4–42 and pyroglutamate Aβ 3–42 in a mouse model of Alzheimer’s disease. Front. Aging Neurosci. 10:64. 10.3389/fnagi.2018.00064 29568268 PMC5852075

[B50] Marcello A. Wirths O. Schneider-Axmann T. Degerman-Gunnarsson M. Lannfelt L. Bayer T. A.. (2009). Circulating immune complexes of Aβ and IgM in plasma of patients with Alzheimer’s disease. J. Neural Transm. 116, 913–920. 10.1007/s00702-009-0224-y 19415450 PMC2700872

[B51] Marcello A. Wirths O. Schneider-Axmann T. Degerman-Gunnarsson M. Lannfelt L. Bayer T. A.. (2011). Reduced levels of IgM autoantibodies against N-truncated pyroglutamate Aβ in plasma of patients with Alzheimer’s disease. Neurobiol. Aging 32, 1379–1387. 10.1016/j.neurobiolaging.2009.08.011 19781815

[B52] Masters C. L. Simms G. Weinman N. A. Multhaup G. Mcdonald B. L. Beyreuther K.. (1985). Amyloid plaque core protein in Alzheimer disease and Down syndrome. Proc. Natl. Acad. Sci. 82, 4245–4249. 10.1073/pnas.82.12.4245 3159021 PMC397973

[B53] Mclaurin J. Cecal R. Kierstead M. E. Tian X. Phinney A. L. Manea M.. (2002). Therapeutically effective antibodies against amyloid-β peptide target amyloid-β residues 4–10 and inhibit cytotoxicity and fibrillogenesis. Nat. Med. 8, 1263–1269. 10.1038/nm790 12379850

[B54] Miller D. L. Papayannopoulos I. A. Styles J. Bobin S. A. Lin Y. Y. Biemann K.. (1993). Peptide compositions of the cerebrovascular and senile plaque core amyloid deposits of Alzheimer’s disease. Arch. Biochem. Biophys. 301, 41–52. 10.1006/abbi.1993.1112 8442665

[B55] Mintun M. A. Lo A. C. Duggan Evans C. Wessels A. M. Ardayfio P. A. Andersen S. W.. (2021). Donanemab in early Alzheimer’s disease. N. Engl. J. Med. 384, 1691–1704. 10.1056/NEJMoa2100708 33720637

[B56] Miravalle L. Calero M. Takao M. Roher A. E. Ghetti B. Vidal R.. (2005). Amino-terminally truncated Aβ peptide species are the main component of cotton wool plaques. Biochemistry 44, 10810–10821. 10.1021/bi0508237 16086583

[B57] Mital M. Bal W. Fraczyk T. Drew S. C. (2018). Interplay between copper, neprilysin and N-truncation of β-Amyloid. Inorg. Chem. 57, 6193–6197. 10.1021/acs.inorgchem.8b00391 29774745

[B58] Morgan D. (2011). Immunotherapy for Alzheimer’s disease. J. Intern. Med. 269, 54–63. 10.1111/j.1365-2796.2010.02315.x 21158978 PMC3074967

[B59] Morgan D. Diamond D. M. Gottschall P. E. Ugen K. E. Dickey C. Hardy J.. (2000). Aβ peptide vaccination prevents memory loss in an animal model of Alzheimer’s disease. Nature 408, 982–985. 10.1038/35050116 11140686

[B60] Murayama K. S. Kametani F. Tabira T. Araki W. (2007). A novel monoclonal antibody specific for the amino-truncated β-amyloid Aβ 5–40/42 produced from caspase-cleaved amyloid precursor protein. J. Neurosci. Methods 161, 244–249. 10.1016/j.jneumeth.2006.11.010 17207535

[B61] Näslund J. Schierhorn A. Hellman U. Lannfelt L. Roses A. D. Tjernberg L. O.. (1994). Relative abundance of Alzheimer A β amyloid peptide variants in Alzheimer disease and normal aging. Proc. Natl. Acad. Sci. 91, 8378–8382. 10.1073/pnas.91.18.8378 8078890 PMC44609

[B62] Nath A. Hall E. Tuzova M. Dobbs M. Jons M. Anderson C.. (2003). Autoantibodies to amyloid β-peptide (Aβ) are increased in Alzheimer’s disease patients and Aβ antibodies can enhance Aβ neurotoxicity: implications for disease pathogenesis and vaccine development. Neuromol. Med. 3, 29–39. 10.1385/nmm:3:1:29 12665674

[B63] Oakley H. Cole S. L. Logan S. Maus E. Shao P. Craft J.. (2006). Intraneuronal β-amyloid aggregates, neurodegeneration and neuron loss in transgenic mice with five familial Alzheimer’s disease mutations: potential factors in amyloid plaque formation. J. Neurosci. 26, 10129–10140. 10.1523/JNEUROSCI.1202-06.2006 17021169 PMC6674618

[B64] Pajonk F. Kessler H. Supprian T. Hamzei P. Bach D. Schweickhardt J.. (2005). Cognitive decline correlates with low plasma concentrations of copper in patients with mild to moderate Alzheimer’s disease. J. Alzheimers Dis. 8, 23–27. 10.3233/jad-2005-8103 16155346

[B65] Parodi-Rullan R. Ghiso J. Cabrera E. Rostagno A. Fossati S. (2020). Alzheimer’s amyloid β heterogeneous species differentially affect brain endothelial cell viability, blood-brain barrier integrity and angiogenesis. Aging Cell 19:e13258. 10.1111/acel.13258 33155752 PMC7681048

[B66] Piccini A. Russo C. Gliozzi A. Relini A. Vitali A. Borghi R.. (2005). β-amyloid is different in normal aging and in Alzheimer disease. J. Biol. Chem. 280, 34186–34192. 10.1074/jbc.M501694200 16103127

[B67] Pike C. J. Overman M. J. Cotman C. W. (1995). Amino-terminal deletions enhance aggregation of β-amyloid peptides *in vitro*. J. Biol. Chem. 270, 23895–23898. 10.1074/jbc.270.41.23895 7592576

[B68] Portelius E. Bogdanovic N. Gustavsson M. K. Volkmann I. Brinkmalm G. Zetterberg H.. (2010). Mass spectrometric characterization of brain amyloid β isoform signatures in familial and sporadic Alzheimer’s disease. Acta Neuropathol. 120, 185–193. 10.1007/s00401-010-0690-1 20419305 PMC3568930

[B69] Rijal Upadhaya A. Kosterin I. Kumar S. Von Arnim C. A. Yamaguchi H. Fandrich M.. (2014). Biochemical stages of amyloid-β peptide aggregation and accumulation in the human brain and their association with symptomatic and pathologically preclinical Alzheimer’s disease. Brain 137, 887–903. 10.1093/brain/awt362 24519982

[B70] Rosen R. F. Tomidokoro Y. Farberg A. S. Dooyema J. Ciliax B. Preuss T. M.. (2016). Comparative pathobiology of β-amyloid and the unique susceptibility of humans to Alzheimer’s disease. Neurobiol. Aging 44, 185–196. 10.1016/j.neurobiolaging.2016.04.019 27318146 PMC4913040

[B71] Rufenacht P. Guntert A. Bohrmann B. Ducret A. Dobeli H. (2005). Quantification of the Aβ peptide in Alzheimer’s plaques by laser dissection microscopy combined with mass spectrometry. J. Mass Spectrom. 40, 193–201. 10.1002/jms.739 15706631

[B72] Russo C. Saido T. C. Debusk L. M. Tabaton M. Gambetti P. Teller J. K.. (1997). Heterogeneity of water-soluble amyloid β-peptide in Alzheimer’s disease and Down’s syndrome brains. FEBS Lett. 409, 411–416. 10.1016/s0014-5793(97)00564-4 9224700

[B73] Salloway S. Sperling R. Fox N. C. Blennow K. Klunk W. Raskind M.. (2014). Two phase 3 trials of bapineuzumab in mild-to-moderate Alzheimer’s disease. N. Engl. J. Med. 370, 322–333. 10.1056/NEJMoa1304839 24450891 PMC4159618

[B74] Schenk D. Barbour R. Dunn W. Gordon G. Grajeda H. Guido T.. (1999). Immunization with amyloid-β attenuates Alzheimer-disease-like pathology in the PDAPP mouse. Nature 400, 173–177. 10.1038/22124 10408445

[B75] Schlenzig D. Cynis H. Hartlage-Rubsamen M. Zeitschel U. Menge K. Fothe A.. (2018). Dipeptidyl-peptidase activity of meprin β links N-truncation of Aβ with glutaminyl cyclase-catalyzed pGlu-Aβ formation. J. Alzheimers Dis. 66, 359–375. 10.3233/JAD-171183 30320570

[B76] Selkoe D. J. (2001). Alzheimer’s disease: genes, proteins and therapy. Physiol. Rev. 81, 741–766. 10.1152/physrev.2001.81.2.741 11274343

[B77] Sergeant N. Bombois S. Ghestem A. Drobecq H. Kostanjevecki V. Missiaen C.. (2003). Truncated β-amyloid peptide species in pre-clinical Alzheimer’s disease as new targets for the vaccination approach. J. Neurochem. 85, 1581–1591. 10.1046/j.1471-4159.2003.01818.x 12787077

[B78] Sevalle J. Amoyel A. Robert P. Fournie-Zaluski M. C. Roques B. Checler F.. (2009). Aminopeptidase A contributes to the N-terminal truncation of amyloid β-peptide. J. Neurochem. 109, 248–256. 10.1111/j.1471-4159.2009.05950.x 19187443

[B79] Sichler M. E. Low M. J. Schleicher E. M. Bayer T. A. Bouter Y. (2019). Reduced acoustic startle response and prepulse inhibition in the Tg4–42 model of Alzheimer’s disease. J. Alzheimers Dis. Rep. 3, 269–278. 10.3233/ADR-190132 31867566 PMC6918877

[B80] Sokoloff L. (2008). The physiological and biochemical bases of functional brain imaging. Cogn. Neurodyn 2, 1–5. 10.1007/s11571-007-9033-x 19003468 PMC2289249

[B81] Tekirian T. L. (2001). Commentary: Aβ N- terminal isoforms: critical contributors in the course of AD pathophysiology. J. Alzheimers Dis. 3, 241–248. 10.3233/jad-2001-3209 12214065

[B82] Trieb K. Ransmayr G. Sgonc R. Lassmann H. Grubeck-Loebenstein B. (1996). APP peptides stimulate lymphocyte proliferation in normals, but not in patients with Alzheimer’s disease. Neurobiol. Aging 17, 541–547. 8832628 10.1016/0197-4580(96)00068-1

[B83] Valverde A. Dunys J. Lorivel T. Debayle D. Gay A. S. Lacas-Gervais S.. (2021). Aminopeptidase A contributes to biochemical, anatomical and cognitive defects in Alzheimer’s disease (AD) mouse model and is increased at early stage in sporadic AD brain. Acta Neuropathol. 141, 823–839. 10.1007/s00401-021-02308-0 33881611 PMC8113186

[B84] Vassar R. Bennett B. D. Babu-Khan S. Kahn S. Mendiaz E. A. Denis P.. (1999). β-secretase cleavage of Alzheimer’s amyloid precursor protein by the transmembrane aspartic protease BACE. Science 286, 735–741. 10.1126/science.286.5440.735 10531052

[B85] Wagner J. M. Sichler M. E. Schleicher E. M. Franke T. N. Irwin C. Low M. J.. (2019). Analysis of motor function in the Tg4–42 mouse model of Alzheimer’s disease. Front. Behav. Neurosci. 13:107. 10.3389/fnbeh.2019.00107 31156407 PMC6533559

[B86] Walsh D. M. Klyubin I. Fadeeva J. V. Cullen W. K. Anwyl R. Wolfe M. S.. (2002). Naturally secreted oligomers of amyloid β protein potently inhibit hippocampal long-term potentiation *in vivo*. Nature 416, 535–539. 10.1038/416535a 11932745

[B87] Walter S. Jumpertz T. Huttenrauch M. Ogorek I. Gerber H. Storck S. E.. (2019). The metalloprotease ADAMTS4 generates N-truncated Aβ4-x species and marks oligodendrocytes as a source of amyloidogenic peptides in Alzheimer’s disease. Acta Neuropathol. 137, 239–257. 10.1007/s00401-018-1929-5 30426203

[B88] Weksler M. E. Relkin N. Turkenich R. Larusse S. Zhou L. Szabo P.. (2002). Patients with Alzheimer disease have lower levels of serum anti-amyloid peptide antibodies than healthy elderly individuals. Exp. Gerontol. 37, 943–948. 10.1016/s0531-5565(02)00029-3 12086704

[B89] Wildburger N. C. Esparza T. J. Leduc R. D. Fellers R. T. Thomas P. M. Cairns N. J.. (2017). Diversity of amyloid-β proteoforms in the Alzheimer’s disease brain. Sci. Rep. 7:9520. 10.1038/s41598-017-10422-x 28842697 PMC5572664

[B90] Wirths O. Zampar S. (2019). Emerging roles of N- and C-terminally truncated Aβ species in Alzheimer’s disease. Expert Opin. Ther. Targets 23, 991–1004. 10.1080/14728222.2019.1702972 31814468

[B91] Wirths O. Walter S. Kraus I. Klafki H. W. Stazi M. Oberstein T. J.. (2017). N-truncated Aβ4-x peptides in sporadic Alzheimer’s disease cases and transgenic Alzheimer mouse models. Alzheimers Res. Ther. 9:80. 10.1186/s13195-017-0309-z 28978359 PMC5628465

[B92] Wittnam J. L. Portelius E. Zetterberg H. Gustavsson M. K. Schilling S. Koch B.. (2012). Pyroglutamate amyloid β (Aβ) aggravates behavioral deficits in transgenic amyloid mouse model for Alzheimer disease. J. Biol. Chem. 287, 8154–8162. 10.1074/jbc.M111.308601 22267726 PMC3318696

[B93] Zampar S. Klafki H. W. Sritharen K. Bayer T. A. Wiltfang J. Rostagno A.. (2020). N-terminal heterogeneity of parenchymal and vascular amyloid-β deposits in Alzheimer’s disease. Neuropathol. Appl. Neurobiol. 46, 673–685. 10.1111/nan.12637 32497293 PMC8082844

